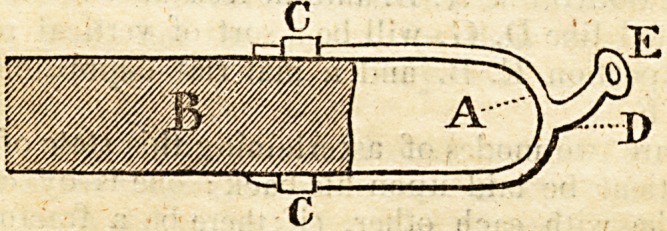# Mr. Harrold on the Treatment of Fractured Thigh

**Published:** 1811-08

**Authors:** E. Harrold

**Affiliations:** Cheshunt, Herts


### *To the Editors of the Medical and Physical Journal.*

GENTLEMEN,

I LATELY sent you the history of a case of fracture of the vertebræ, in which the mode of treatment there detailed was, in a great degree, successful.

I have now a few remarks to offer on the subject of fractures of the thigh, which, as they are the result of further experience, may be useful to my professional brethren and their patients; and, not the less so, for their being sufficiently obvious when pointed out, if they have escaped particular notice before.

It has long been the established practice, I believe, in fractures of the thigh (except in those cases where the fracture has been supposed to be within the capsular ligament at its upper extremity) to keep the limb in a bent position, whether the patient lay upon his back or upon his side. This has been my own practice: but there is a circumstance, which I am about to explain, of considerable importance, and which, I believe, has not hitherto been observed.

The objects of the Surgeon in setting a fracture of this limb are, of course, as much as possible to prevent the twisting of the the bone, if I may be allowed that expression, and to preserve its length. To secure the latter object, the plan first proposed by Mr. Pott, of placing the limb in such a position as to relax the muscles, generally, surrounding it, has been adopted.

Now, supposing the fracture to be within a few inches of the knee joint, the success of this treatment will materially depend upon the *direction* of the fracture; for, if the fracture be transverse, or if oblique, and the direction of that obliquity be not such that the *lower* fractured portion may rest *upon* the *upper* fractured portion, when the knee is bent, as much as it usually is on these occasions, the large body of muscle attached to the patella, will, by its communicated action upon the extremity of the bone, occasion a very material depression, and consequent separation of the lower fractured portion from the upper.

Let the parallel lines A. B. C. D. represent the os femoris— A. B. its anterior, C. D. its posterior surface when in an erect position; A. C. its upper, and B. D. its lower extremity. Let the line E. F. represent the oblique fracture of that bone in the direction (still in relation to its upright position) upwards and backwards. Let the dotted line from A. to B. represent the extensor muscles of the leg attached to the patella at B; and let the dotted line D. G. represent the direction of the resisting power, as applied to the calf of the leg, when *bent* at the knee, to prevent the shortening of the distance between A. and B.—the result of the action of the muscles of the dotted line A. B. and the resistance in the direction of the dotted line D. G. will be a sort of vertical rotation of the lower portion E. B. and a material depression of it at the point F.

There are two modes of ascertaining this state of the case, if the patient be laid upon his back; one is by comparing the patellæ with each other, (if there be a fracture of one thigh only) the patella of the fractured limb will be found, in proportion to the proximity of the fracture to the joint, to be evidently *less prominent* than the other. The other mode is, by passing the fingers along the under surface of the fracture, when the depression will be evident to the touch.

If, however, the patella of the fractured limb be as prominent as the other, the Surgeon need make no farther inquiry, as he may then depend upon the under portion of bone being properly supported by the upper.

Having pointed out this distinction, it is scarcely necessary for me to remark that, where the fracture is transverse, or where the obliquity, as represented in the diagram, is upwards and backwards, the limb must be very little bent at the knee, and the rectus muscle would be still more relaxed if the body could be in some degree bent upon the thigh. I am not aware of any mode of preserving the proper length of the limb, where some resistance to the contraction of the muscles is not made by pressure upon the calf of the leg, and, when patients are placed upon the fracture machine, this is the only part which suffers pain. The cushion must therefore be made of the softest materials; ease may be frequently obtained by lessening the pressure upon particular points, insinuating for that purpose small portions of wool on each side, upon the end of a spatula; and the less the knee is bent, admitting that it is sufficiently so to answer the purpose, the less will the patient suffer from the pressure.

A new, but untried expedient occurs to me, which I am disposed to think may answer the purpose, and would deserve trial in the event of failure of the plan I have just mentioned—it is this—Let there be a band of stout buck skin, or buff leather (which last has the united qualities of firmness and softness, and might, if requisite, have a padding of wool on its inner surface.) Let there be a band of this description, about two inches broad, buckled, moderately tight, above the knee at the usual gartering place, with the buckle on one side, and the skin beneath it protected by a prolongation of the strap made rather wider than the buckle. To the front edge of this band, immediately above the patella, let the end of a strap be fixed.

Then let an iron A. somewhat in the form of a spur, be projected from the knee extremity of the fracture-box B (see the plate in vol. XV facing p. 16.) to which it may be fixed, when wanted, by its also sliding into the staples C C, and what corresponds with the shank of the spur D should be curved upwards and have a loop at the extremity E, to which a buckle should be fixed for the strap from the band to be led through; through; by which means a moderate degree of extending power might be employed, so as to *counteract* the injurious action of the muscles attached to the patella.

It must be remembered that, as only a *moderate* degree of extension in this way could probably be borne, this must be considered only as an *auxiliary* power, and employed when the action of the muscles is *almost* prevented by the limb being but little bent at the knee.

I am, Gentlemen,
Your humble Servant,
E. HARROLD.

*Cheshunt, Herts, May* 14, 1811.

## Figures and Tables

**Figure f1:**
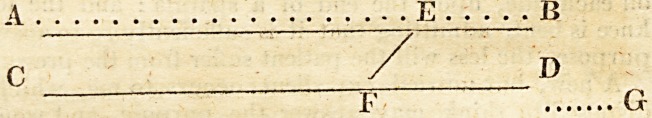


**Figure f2:**